# Supplementary material for: Large-scale drug sensitivity, gene dependency, and proteogenomic analyses of telomere maintenance mechanisms in cancer cells
Source: Nat Commun. 2025 Dec 23;16:11337. doi: 10.1038/s41467-025-67190-w (PMC12727880; doi:10.1038/s41467-025-67190-w)
Supplement: Supplementary file 1 — Description of Additional Supplementary Information [file 41467_2025_67190_MOESM1_ESM.pdf]

Description of Additional Supplementary Information

Supplementary Data 1
Cell line annotations, proliferation and suppliers.

Supplementary Data 2
Telomere biology assay results for 976 human cancer cell lines.

Supplementary Data 3
Multi-omic annotation of ATRX/DAXX/TERT/TERC genes in 91 cancer cell lines (relating to Figure 4).

Supplementary Data 4
Prediction outputs for ALT and TA prediction algorithms (relating to Figure 5).

Supplementary Data 5
proteotranscriptomic analysis of telomere maintenance mechanism and telomerase activity levels (relating to Figure 6 and S5B).

Supplementary Data 6
Gene dependency analysis of telomere maintenance mechanism, telomerase activity levels and telomere content (relating to Figure 7).

Supplementary Data 7
Drug sensitivity analysis of telomere maintenance mechanism and telomerase activity levels (relating to Figure 8).
