# Supplementary material for: Large-scale drug sensitivity, gene dependency, and proteogenomic analyses of telomere maintenance mechanisms in cancer cells
Source: Nat Commun. 2025 Dec 23;16:11337. doi: 10.1038/s41467-025-67190-w (PMC12727880; doi:10.1038/s41467-025-67190-w)
Supplement: Supplementary file 9 — Reporting Summary [file 41467_2025_67190_MOESM9_ESM.pdf]

nature portfolio

Corresponding author(s): Karen MacKenzie
Roger Reddel
Qing Zhong

Last updated by author(s): Nov 22, 2025

Reporting Summary

Nature Portfolio wishes to improve the reproducibility of the work that we publish. This form provides structure for consistency and transparency in reporting. For further information on Nature Portfolio policies, see our Editorial Policies and the Editorial Policy Checklist.

Statistics

For all statistical analyses, confirm that the following items are present in the figure legend, table legend, main text, or Methods section.

n/a	Confirmed	
☐	☒	The exact sample size (*n*) for each experimental group/condition, given as a discrete number and unit of measurement
☐	☒	A statement on whether measurements were taken from distinct samples or whether the same sample was measured repeatedly
☐	☒	The statistical test(s) used AND whether they are one- or two-sided<br>*Only common tests should be described solely by name; describe more complex techniques in the Methods section.*
☐	☒	A description of all covariates tested
☐	☒	A description of any assumptions or corrections, such as tests of normality and adjustment for multiple comparisons
☐	☒	A full description of the statistical parameters including central tendency (e.g. means) or other basic estimates (e.g. regression coefficient) AND variation (e.g. standard deviation) or associated estimates of uncertainty (e.g. confidence intervals)
☐	☒	For null hypothesis testing, the test statistic (e.g. *F*, *t*, *r*) with confidence intervals, effect sizes, degrees of freedom and *P* value noted<br>*Give P values as exact values whenever suitable.*
☒	☐	For Bayesian analysis, information on the choice of priors and Markov chain Monte Carlo settings
☒	☐	For hierarchical and complex designs, identification of the appropriate level for tests and full reporting of outcomes
☐	☒	Estimates of effect sizes (e.g. Cohen's *d*, Pearson's *r*), indicating how they were calculated

Our web collection on statistics for biologists contains articles on many of the points above.

Software and code

Policy information about availability of computer code

Data collection	The PharmacoGx package within the R software environment (version 4.4.1) was used to collect drug response data of CTRP and PRISM data sets. Image acquisition for immunofluorescence employed ZEN software (version 3.9.101.03000, Zeiss). Phosphor imaging data from C-Circle and TRF assays was collected using Amersham™ Typhoon™ control software, version 3.0.0.2, Cytiva), Western blot images were visualized using the GE ImageQuant™ (version 8.1.0.0, Fujifilm). Growth curves for siRNA suppression experiments were generated using the Incucyte® ZOOM system (version 2018A).
Data analysis	R (version 4.2.3), Python (version 3.10.11), Python package SHapley Additive exPlanations (SHAP) (v0.41.0), and GraphPad Prism (version 10..1) were employed for data analysis. CellProfiler version 4.2.5 was used for image analysis; densitometric data was analyzed using ImageQuant software (version version 8.1.0.0, Molecular Dynamics). Proteomic data were processed using DIA-NN version 1.8.1; whole genome sequencing data were analyzed using the nf-core/sarek pipeline version 3.4.0 implemented in Nextflow version 23.10.1; Aadapter trimming and quality control were performed using FastP version 0.23.4 and FastQC version 0.12.1; read alignment was done using bwa-mem version 0.7.17-r1188; variant calling employed GATK version 4.4.0.0; structural variants were identified using Manta, Smoove, and TIDDIT, and merged using SURVIVOR. AnnotSV was used for annotation, and Samplot for visualization, copy number variation analysis was conducted using cnvkit version 0.9.10; Gene set enrichment and over-representation analyses were performed using the WebGestalt 2019 tool; sequential forward selection (SFS) was implemented using the Python package mlxtend version 0.20; multinomial logistic regression and multiple linear regression models were developed using scikit-learn version 1.1.2, and feature importance was calculated using SHAP version 0.41.0. Statistical analyses were performed using the Stats, ROCR and ggpubr packages in R version 4.4.1, and GraphPad Prism version 10.6.1.<br><br>Code used for data analysis has been deposited in Zenodo: [https://doi.org/10.5281/zenodo.16892621].

For manuscripts utilizing custom algorithms or software that are central to the research but not yet described in published literature, software must be made available to editors and reviewers. We strongly encourage code deposition in a community repository (e.g. GitHub). See the Nature Portfolio guidelines for submitting code & software for further information.

Data

Policy information about availability of data

All manuscripts must include a data availability statement. This statement should provide the following information, where applicable:

- Accession codes, unique identifiers, or web links for publicly available datasets
- A description of any restrictions on data availability
- For clinical datasets or third party data, please ensure that the statement adheres to our policy

A data availability section is included in the Methods on page 24 of the manuscript. This section includes the identifier and link to our proteomic and genomic matrices deposited in PRIDE and SRA database respectively. This data is now publicly available. Access to external datasets is via publicly available portals or publications that are identified in our Data Availability Statement, other sections of the Methods and Figure Legends. Hyperlinks and citations are provided in the Data Availability section for external data sets. There are no restrictions on data availability or use; All other data are provided in the article Supplementary Information or Source Data.

Measurements from TMM assays, including qTRAP, C-Circle assay, TC qPCR, ALT-FISH, APB assays, Telomere-FISH, TC by qPCR, and Western blot analysis of ATRX/ DAXX are provided in Supplementary Data 2. Raw and processed proteomic data generated in this study and accompanying files have been deposited in the ProteomeXchange Consortium via the PRIDE partner repository with the dataset identifier PXD058664 [https://www.ebi.ac.uk/pride/archive/projects/PXD058664]. Whole genome sequencing data generated from 14 ALT and ALT-Low cell lines were deposited in the Sequence Read Archive (SRA) database under accession code PRJNA1251703 [https://www.ncbi.nlm.nih.gov/sra/?term=PRJNA1251703]. Summarized data on ATRX and DAXX mutations, SVs and CNVs are included in Supplementary Information (Supplementary Data 3).

Genomic and transcriptomic data for other cell lines were retrieved from external sources: SNV and indels (version 20221018), gene fusion (version 20191101), CNV (version 20221213), and RNA expression (version 20220624) were downloaded from Cell Models Passport [https://cellmodelpassports.sanger.ac.uk/downloads]10. SV data (translocation SvABA version CCLE 2019) was also obtained from DepMap [https://depmap.org/portal/]9. Telomere content data, previously derived from whole exome sequencing and whole genome sequencing, were downloaded from DepMap [https://depmap.org/portal/data_page/?tab=allData]12. TERT promoter mutations and THOR methylation were retrieved from previous publications 9, 113. Gene dependency data [version: Integrated CERES_ComBat+QN+PC1] was downloaded from Figshare [https://figshare.com/articles/dataset/Integrated_Merged_Datasets/13252640?file=25521821]29. The GDSC drug screen datasets were previously published [https://figshare.com/articles/dataset/Pan-cancer_proteomic_map_of_949_human_cell_lines/19345397?file=34355645]13. CTRP (version CTRPv2_2015) and PRISM (version PRISM_2020) drug screen results were downloaded from PharmacoDB [https://pharmacodb.ca]117 using the PharmacoGx package in R (version 4.4.1). Data used for validation of TMM/TA predictors were from the following sources: GEO Series accession number GSE14533[https://www.ncbi.nlm.nih.gov/geo/query/acc.cgi?acc=GSE14533]21, 22 for RNA expression in human cell lines, hMSCs and the liposarcoma tissue cohort; DepMap [https://depmap.org/portal/](version 22Q4) for RNA expression in the CCLE lung and bladder cell lines 23, 24; and a previous publication for RNA expression data from Glioma sphere-forming cells and EXTEND Score data 23. The remaining data are available within the Article, Supplementary Information or Source Data file.

Research involving human participants, their data, or biological material

Policy information about studies with human participants or human data. See also policy information about sex, gender (identity/presentation), and sexual orientation and race, ethnicity and racism.

Reporting on sex and gender	This study presents data on a set of 976 cell lines. The cell line panel was based on the availability of associated external data sets, such as 'omics, drug sensitivity and gene dependencies. Sex was not a consideration in the selection of cell lines for this panel. The cell line panel covers approximately 60 different cancer types, encompassing cancers that affect males, females and both sexes. Where the information is available, the sex of the patient from which the cell line was derived is provided in Table S1. The ratio of cell lines from males to females is 1.00:0.73, which approximates the higher incidence of cancer in men.
Reporting on race, ethnicity, or other socially relevant groupings	Information on race, ethnicity and social groupings have not been reported as the available data is not comprehensive enough.
Population characteristics	The cell lines were originally derived from male and female children and adults, in the age bracket of 0 yrs to 89 yrs. This information is provided in Table S1 where available.
Recruitment	This study employed previously established cell lines. No additional subjects were recruited.
Ethics oversight	This study was conducted in accordance with governance and ethical oversight policies in place at Children's Medical Research Institute.

Note that full information on the approval of the study protocol must also be provided in the manuscript.

Field-specific reporting

Please select the one below that is the best fit for your research. If you are not sure, read the appropriate sections before making your selection.

- [x] Life sciences
- [ ] Behavioural & social sciences
- [ ] Ecological, evolutionary & environmental sciences

For a reference copy of the document with all sections, see nature.com/documents/nr-reporting-summary-flat.pdf

Life sciences study design

All studies must disclose on these points even when the disclosure is negative.

Sample size	976 cell lines
Data exclusions	Data from cell lines that did not pass identity tests by STR profiling were excluded. There were no other data exclusions.
Replication	All telomere biology assays were performed as replicates. As shown in the manuscript, orthogonal tests were performed to verify classifications based on telomere biology data. Additionally, to ensure reproducibility, we performed telomere biology assays on a a large representative subset of the cohort using cell lines from independent sources and grown in an independent laboratory. Comparisons of the results from samples grown in independent laboratories are provided in the Supplementary Information. Technical and biological replicates were not conflated; average values were taken from technical replicates before the mean of biological replicates was calculated. This has been clarified in the revised legend for Figure 3A and in the Statistics and Reproducibility section.
Randomization	Wet-lab tests were performed in batches based on time of arrival to our lab<br>Samples for proteomic analysis were processed in randomised batches. This is reported in the he Statistics and Reproducibility section.
Blinding	Since there was no existing data on the parameters measured for most of the cell lines to provide bias, most experiments were performed unblinded, with exception of orthogonal validation of TA in Figure S1A and mass spectrometry which were performed blinded. This is reported in the he Statistics and Reproducibility section.

Reporting for specific materials, systems and methods

We require information from authors about some types of materials, experimental systems and methods used in many studies. Here, indicate whether each material, system or method listed is relevant to your study. If you are not sure if a list item applies to your research, read the appropriate section before selecting a response.

Materials & experimental systems

n/a	Involved in the study	
☐	☒	Antibodies
☐	☒	Eukaryotic cell lines
☒	☐	Palaeontology and archaeology
☒	☐	Animals and other organisms
☒	☐	Clinical data
☒	☐	Dual use research of concern
☒	☐	Plants

Methods

n/a	Involved in the study	
☒	☐	ChIP-seq
☐	☒	Flow cytometry
☒	☐	MRI-based neuroimaging

Antibodies

Antibodies used

Anti-ATRX, 1:400 dilution, Sigma, Cat#HPA001906, Lot# 000021633 RRID:AB_1078249;
Anti-DAXX, 1:750 dilution, Sigma, Cat#HPA008736, Lot#BG117554, RRID:AB_1078625;
Anti-Actin, 1:500 dilution, Sigma, Cat#A2066, Lot#106M4770V, RRID:AB_476693;
Anti-SAMHD1, 1:500 dilution, Origene, Cat#TA502024, Lot#F001, RRID:AB_11124442;
Goat Anti-Rabbit Immunoglobulins/HRP, 1:5000 dilution, Agilent, Cat#P0448, Lot# 00058739, RRID:AB_2617138;
Goat Anti-Mouse Immunoglobulins/HRP, 1:5000 dilution, Agilent, Cat# P0447
Goat Anti-PML Antibody (N-19), 1:500 dilution, Santa Cruz Biotechnology, Cat#sc-9862, Lot# EO613, RRID:AB_2166847;
Alexa Fluor® 488 donkey anti-goat IgG (H+L), 1:500 dilution, Thermo Fisher Scientific,Cat#A32814, Lot# XH354849, RRID:AB_2762838;
Anti-hTERT sheep polyclonal, 20 ug/mL, South Australia Health and Medical Research Institute, (Tomlinson et, al., Methods 114; 85-95, 2017)

Validation

Antibodies for ATRX, DAXX, Actin, SAMHD1, PML and secondary antibodies were validated by the commercial provider. Information is available on the websites. These antibodies have also been validated in previous studies listed below:

ATRX and DAXX: de Nonneville A, et al. TOP3A amplification and ATRX inactivation are mutually exclusive events in pediatric osteosarcomas using ALT. EMBO Mol Med. 3, (2022).
PML: Lu, R. et al. The FANCM-BLM-TOP3A-RMI complex suppresses alternative lengthening of telomeres (ALT). Nat Commun 10, 2252 (2019).
SAMHD1: Kapoor-Vazirani P, et al. SAMHD1 deacetylation by SIRT1 promotes DNA end resection by facilitating DNA binding at double-strand breaks. Nat Commun. 19(1):81-89. 2022.
hTERT: Tomlinson, C.G., Sasaki, N., Jurczyluk, J., Bryan, T.M. & Cohen, S.B. Quantitative assays for measuring human telomerase activity and DNA binding properties. Methods 114, 85-95. 2017..

For internal validation in our study, positive and negative controls were included in all all assays that employed these antibodies. These controls are shown in the Figures and Supplementary data as follows:

Figure 2 and S3: U2OS and HeLa - positive and negative controls respectively for PML antibody
Figure 4 and S4: U2OS and HeLa - negative and positive controls respectively for ATRX; HeLa and G292- positive and negative controls respectively for DAXX.
Figure S6: Scrambled control serves as a positive control for each cell line.
The hTERT anti-body was developed in-house and was extensively characterized in Tomlinson et, al., Methods 114; 85-95, 2017 and Cohen et al., Nat. Methods 5; 355-360, 2008. It was used at a concentration of 20 ug/mL. Validation included immunoprecipitation of (a) telomerase (quantified in an enzyme assay) and (b) the telomerase RNA component (TERC) measured by Northern blot. The hTERT antibody was tested in dilution series. A549 was used as a positive control.

Eukaryotic cell lines

Policy information about cell lines and Sex and Gender in Research

Cell line source(s)	Table S1 lists all cell lines, with sex, age and source data.
Authentication	Cell lines were authenticated by 16-locus short-tandem-repeat (STR) profiling at CellBank Australia. This information is provided in the "Ethics and Inclusion" section of Methods.
Mycoplasma contamination	Cell lines were tested for mycoplasma contamination at CellBank Australia. This information is provided in the "Ethics and Inclusion" section of Methods.
Commonly misidentified lines (See ICLAC register)	Cell lines listed in the ICLAC Register v13 (17 October 2025) are indicated in Table S1. Each of these 15 cell lines were authenticated against the expected STR profile. A statement has been added to the Ethics and Inclusion section to refer to this data in Table S1.

Plants

Seed stocks	*Report on the source of all seed stocks or other plant material used. If applicable, state the seed stock centre and catalogue number. If plant specimens were collected from the field, describe the collection location, date and sampling procedures.*
Novel plant genotypes	*Describe the methods by which all novel plant genotypes were produced. This includes those generated by transgenic approaches, gene editing, chemical/radiation-based mutagenesis and hybridization. For transgenic lines, describe the transformation method, the number of independent lines analyzed and the generation upon which experiments were performed. For gene-edited lines, describe the editor used, the endogenous sequence targeted for editing, the targeting guide RNA sequence (if applicable) and how the editor was applied.*
Authentication	*Describe any authentication procedures for each seed stock used or novel genotype generated. Describe any experiments used to assess the effect of a mutation and, where applicable, how potential secondary effects (e.g. second site T-DNA insertions, mosiacism, off-target gene editing) were examined.*

Flow Cytometry

Plots

Confirm that:

- [x] The axis labels state the marker and fluorochrome used (e.g. CD4-FITC).
- [x] The axis scales are clearly visible. Include numbers along axes only for bottom left plot of group (a 'group' is an analysis of identical markers).
- [x] All plots are contour plots with outliers or pseudocolor plots.
- [x] A numerical value for number of cells or percentage (with statistics) is provided.

Methodology

Sample preparation	Cell lines were suspended in 2% FBS in PBS at 2-5 x10E+6 cells/ml for single-cell sorting. DAPI was added at 100ng/ml to exclude dead cells that take up this dye.
Instrument	Becton Dickinson Influx
Software	BD FACS Sortware V1.2.0.142
Cell population abundance	The percentage of viable single cells in the starting population varied from 94.9% (SNU387), 42.1% (SCH) and 65.6% (PANC-09-13). Single cells were sorted into individual wells in a 96-well plate using the singlet gate and single sort mode to ensure only single cells were deposited. A dilution series was dispensed, and wells were visually scanned under an inverted microscope for quality control.
Gating strategy	DAPI-negative Viable cells were gated on SSC v FSC then singlets selected as trigger pulse-width low as shown in Figure S2A.

- [x] Tick this box to confirm that a figure exemplifying the gating strategy is provided in the Supplementary Information.
