# Supplementary material for: Large-scale drug sensitivity, gene dependency, and proteogenomic analyses of telomere maintenance mechanisms in cancer cells
Source: Nat Commun. 2025 Dec 23;16:11337. doi: 10.1038/s41467-025-67190-w (PMC12727880; doi:10.1038/s41467-025-67190-w)

Figure S1

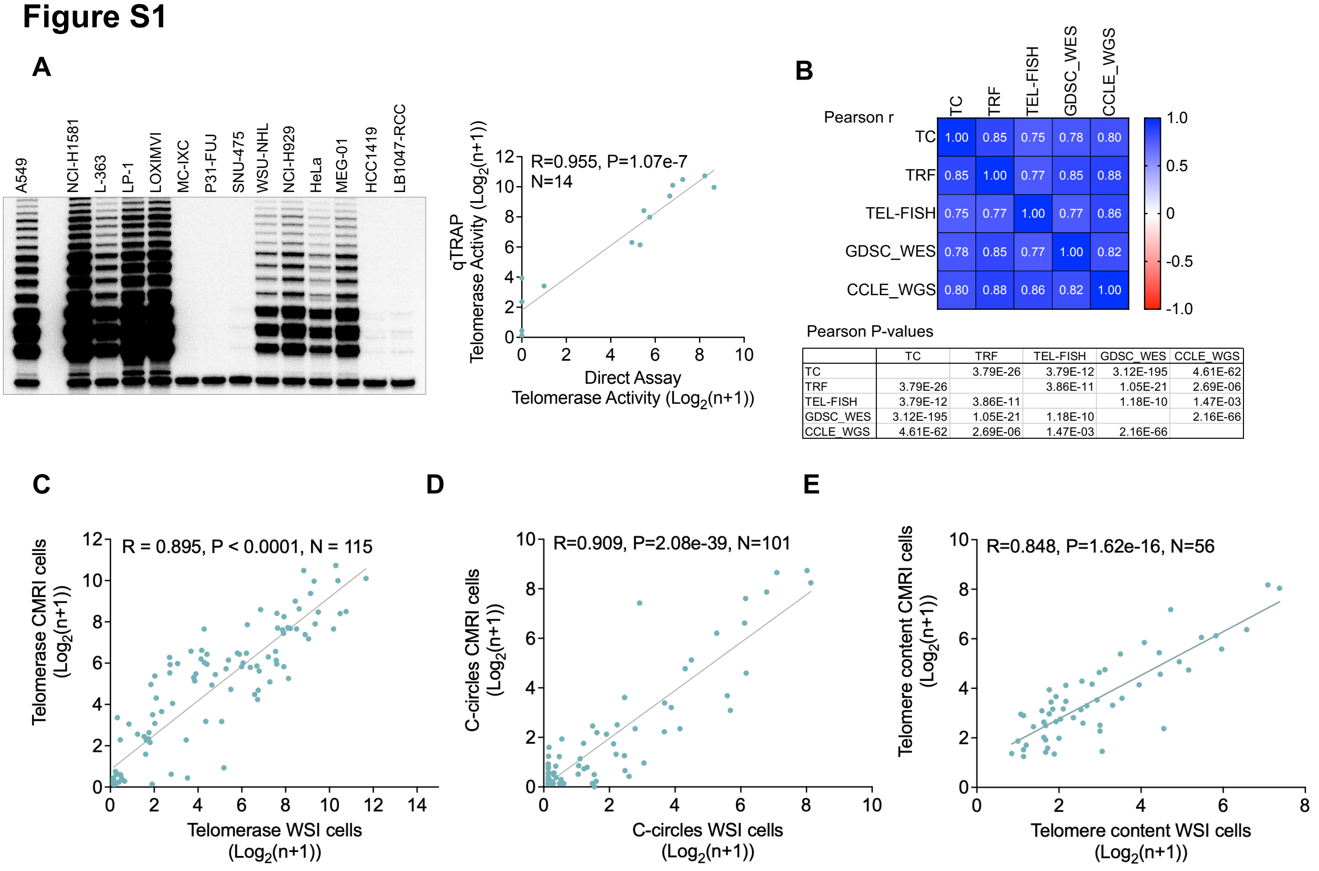

	TC	TRF	TEL-FISH	GDSC_WES	CCLE_WGS
TC		3.79E-26	3.79E-12	3.12E-195	4.61E-62
TRF	3.79E-26		3.86E-11	1.05E-21	2.69E-06
TEL-FISH	3.79E-12	3.86E-11		1.18E-10	1.47E-03
GDSC_WES	3.12E-195	1.05E-21	1.18E-10		2.16E-66
CCLE_WGS	4.61E-62	2.69E-06	1.47E-03	2.16E-66	

Figure S1. Validation of telomere maintenance mechanism (TMM) measurements

(A) Validation of qTRAP data range using a direct primer-extension assay for telomerase activity (TA). A representative gel is shown. Similar results were obtained from a repeat assay using cells collected from an independent time point. **(B)** Correlation between telomere length measurements derived via different methods. Input data are log-transformed telomere content (TC) measured by qPCR (TC, N=976), mean telomere length measured by terminal restriction fragment (TRF) Southern blot analysis (N=89), quantitation of telomeric DNA by fluorescence-in situ hybridization (FISH) to a telomere probe (TEL-FISH, N=60), telomere repeats calculated from whole exome sequencing (WES)(N=946) available from Cell Models Passport (GDSC_WES, N=946) and whole genome sequencing (WGS) available through Cancer Cell Line Encyclopedia (CCLE_WGS, N=272). Data from TC, TRF, and TEL-FISH are in Supplementary Data 2. TC measures from WES and WGS are available through DepMap [https://depmap.org/portal/data_page/?tab=allData]. **(C-E)** Correlation between results from cells cultured independently at Children's Medical Research Institute (CMRI) and Wellcome Sanger Institute (WSI) assayed by qTRAP **(C)**, C-circle (CC) assay **(D)** and qPCR for TC **(E)**. R and P-values were derived from two-sided Pearson's correlation analysis.

Figure S2

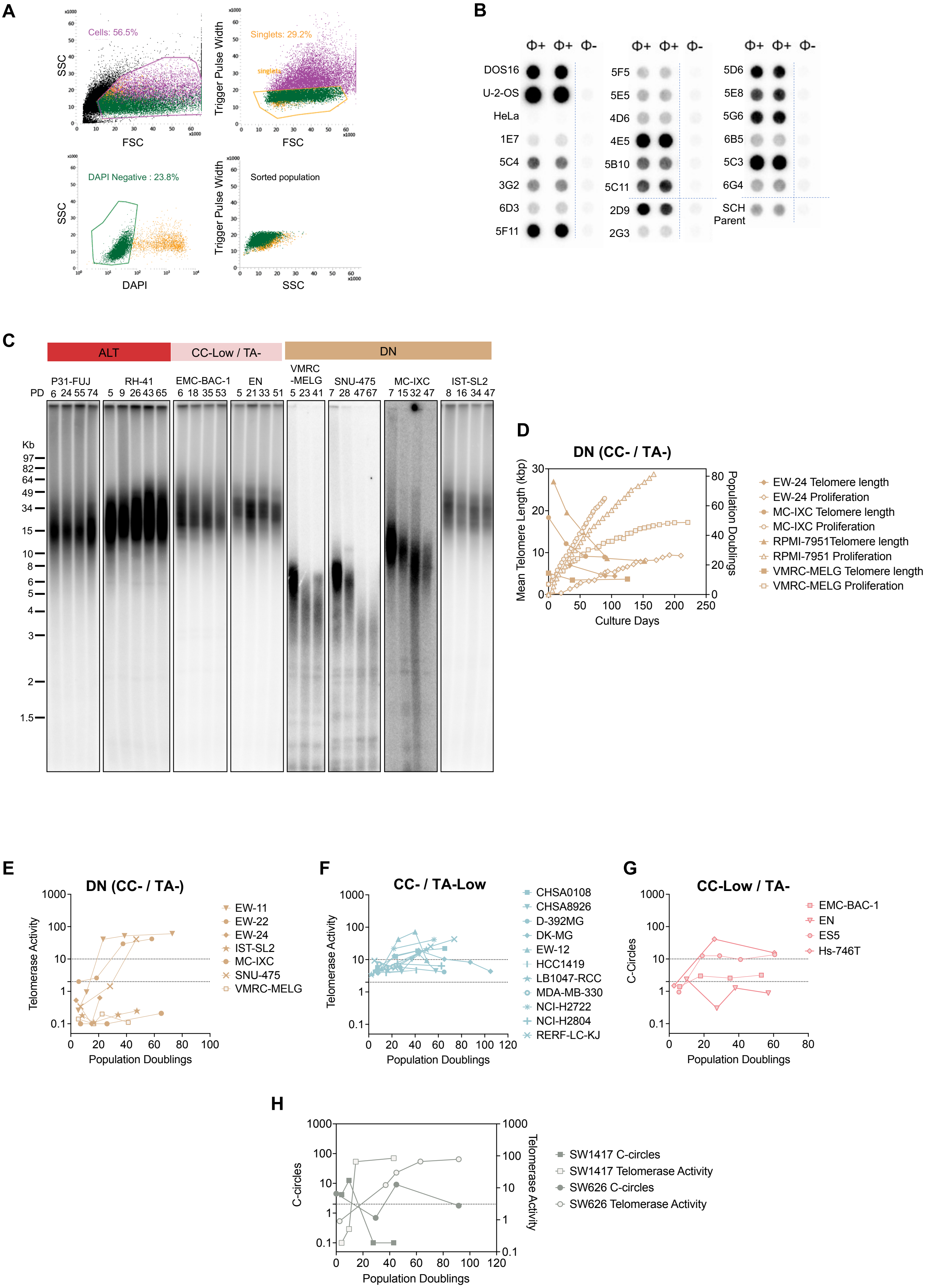

A
Cells: 56.5%
SSC
FSC
Trigger Pulse Width
Singlets: 29.2%
DAPI Negative : 23.8%
DAPI
Sorted population
B
Φ+ Φ+ Φ-
DOS16
U-2-OS
HeLa
1E7
5C4
3G2
6D3
5F11
5F5
5E5
4D6
4E5
5B10
5C11
2D9
2G3
5D6
5E8
5G6
6B5
5C3
6G4
SCH Parent
C
ALT
CC-Low / TA-
DN
P31-FUJ
RH-41
EMC-BAC-1
EN
VMRC -MELG
SNU-475
MC-IXC
IST-SL2
PD
Kb
D
DN (CC- / TA-)
Mean Telomere Length (kbp)
Population Doublings
Culture Days
EW-24 Telomere length
EW-24 Proliferation
MC-IXC Telomere length
MC-IXC Proliferation
RPMI-7951Telomere length
RPMI-7951 Proliferation
VMRC-MELG Telomere length
VMRC-MELG Proliferation
E
DN (CC- / TA-)
Telomerase Activity
Population Doublings
EW-11
EW-22
EW-24
IST-SL2
MC-IXC
SNU-475
VMRC-MELG
F
CC- / TA-Low
CHSA0108
CHSA8926
D-392MG
DK-MG
EW-12
HCC1419
LB1047-RCC
MDA-MB-330
NCI-H2722
NCI-H2804
RERF-LC-KJ
G
CC-Low / TA-
C-Circles
EMC-BAC-1
EN
ES5
Hs-746T
H
C-circles
Telomerase Activity
SW1417 C-circles
SW1417 Telomerase Activity
SW626 C-circles
SW626 Telomerase Activity

Figure S2. Cell lines deviating from canonical Alternative Lengthening of Telomeres (ALT) and telomerase-positive (TEL) phenotypes.

(A) Gating strategy for single-cell sorting of telomere maintenance mechanism (TMM)-double positive (DP) cells by flow cytometry. Single cells were gated using the FSC and SSC parameters versus trigger pulse width. Dead cells were excluded using DAPI stain. qTRAP and CC results from clones derived from sorted single cells are shown in Figure 2A. **(B)** Representative CC blot of 19 subclones established as single cells from the TMM-DP cell line SCH. All subclones and the parent culture were CC+ (CC>2%). Φ+: treated with Φ29 DNA polymerase; Φ-: negative control with no Φ29 DNA polymerase. Dashed lines indicate where the image was rearranged for presentation purposes. Consistent results were obtained from analysis of samples from an independent time point. Quantifications from the two time points are shown in Figure 2A. **(C)** Terminal restriction fragment (TRF) Southern blot analysis of 8 representative cell lines in long-term culture. In total, 38 cell lines were assayed at least 3 time points at increments of at least 10 population doublings (PD). In total 16 TRF Southern blots were performed to quantify the telomere length changes depicted in Figures 2B and S2D, and Supplementary Data 2. Uncropped images are at the end of this file. **(D)** Proliferation and telomere shortening in TMM-Double Negative (DN) cell lines. **(E-F)** TA at sequential time points determined by qTRAP analysis of representative cell lines that were initially CC-/TA- (TA and CCs both <2% control) **(E)**, or CC-/TA-Low (TA in the range of 2%-10% control) **(F)**. **(G)** CCs at sequential time points in TA- cell lines with fluctuating low CCs. **(H)** CCs (left axis) and TA (right axis) at sequential time points in two cell lines that exhibited concurrent changes in TA and CCs.

Figure S3

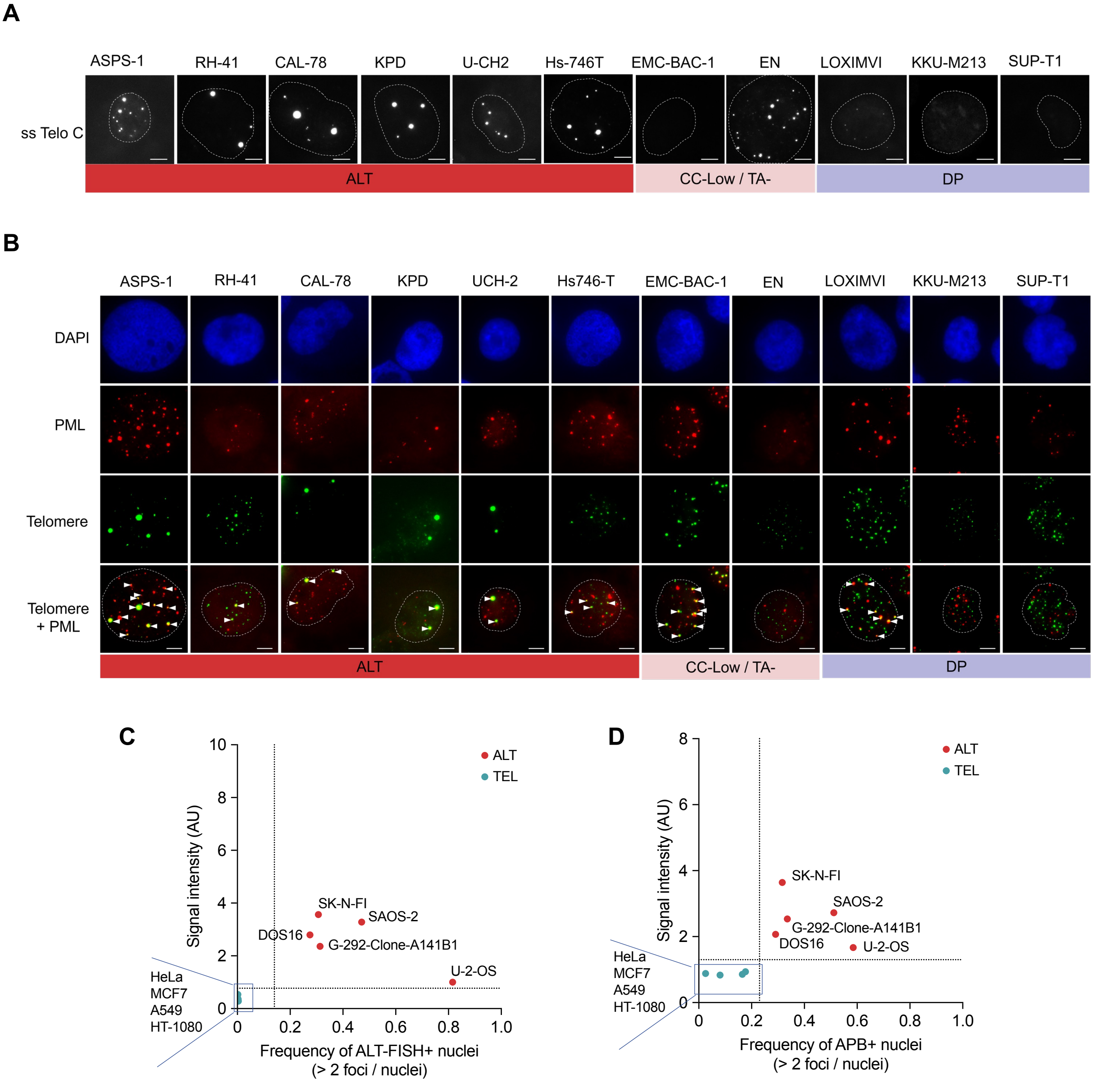

Figure S3. ALT-FISH and ALT-associated PML bodies (APB) assays confirm ALT characteristics in CC-Low/TA- and DP cell lines
(A) Images from ALT-FISH assays showing foci of single-stranded C-rich DNA in cell lines representing various TMM states. **(B)** Images from immunofluorescence staining for ALT-associated PML bodies (APBs), indicated by arrowheads in representative cell lines. **(C)** ALT-FISH results for control ALT and TEL cell lines used to calibrate assays in Figure 2C. **(D)** Results from APB assays for control ALT and TEL cell lines used to calibrate assays in Figure 2D. Scale bars in (A) and (B) are 5μm. Quantifications in (C-D) use data from at least 500 nuclei per cell line. Horizontal and vertical lines in (C) and (D) indicate mid-points between the highest values for control TEL cell lines and the lowest value for control ALT cell lines.

Figure S4

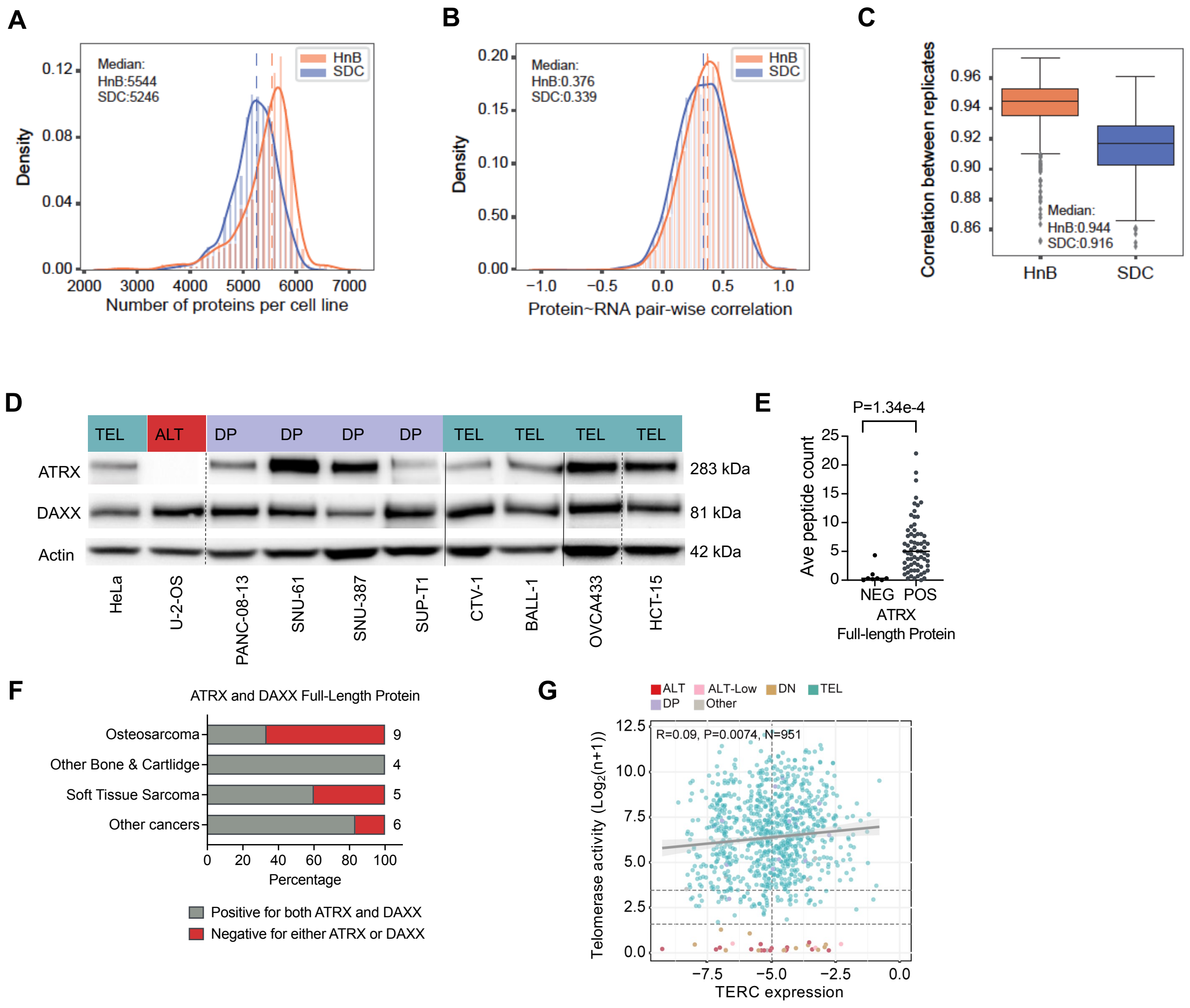

Figure S4: Molecular analysis of ALT and TEL

(A-C) Data-independent acquisition-mass spectrometry (DIA-MS) was performed on 940 cell lines following sample preparation using the Heat and Beat (HnB) protocol. Data was analyzed via the DIA-NN pipeline and compared to previously published results generated using a different method (SDC) (Goncalves et al., 2022). **(A)** Comparison of the number of proteins detected in 916 cell lines common to the HnB and SDC datasets. **(B)** Pairwise correlations between protein and RNA intensities in 916 cell lines common to both datasets. **(C)** Correlations between technical replicates (N=912 cell lines with technical replicates). Boxplots show the 25th and 75th percentiles (box edges), median (horizontal bar), and error bars representing 1.5x interquartile range. **(D)** Representative Western blot analysis showing expression of full-length ATRX and DAXX in 4 representative TMM-DP cell lines and 4 TEL cell lines. HeLa and U-2-OS were used as positive and negative controls, respectively, for ATRX expression. Actin was used as a loading control. ATRX/DAXX A total of 78 cell lines representing different TMM phenotypes were analyzed across 16 independent Western blots. Cell lines were assayed 1-5 times. Additional results are shown in Figure 4A and are summarized in Figure 4B and Supplementary Data 2. Solid vertical lines distinguish independent gels, and dashed lines indicate where lanes were rearranged or excised for presentation purposes. Uncropped source images for this figure are shown at the end of this file. **(E)** Number of ATRX peptides detected by DIA-MS in cell lines assayed for ATRX expression by Western blot analysis. NEG; negative (N=8) and POS; positive (N=68) for detection of full-length ATRX protein. The horizontal line indicates the median value. P-value is from two-sided Wilcoxon rank sum test. **(F)** Cancer type distribution of ATRX and DAXX abnormalities detected by Western blot analysis of ALT and ALT-Low cell lines. The numbers on the right indicate the number of ALT and ALT-Low cell lines per category. Data is summarized in Supplementary Data 2. **(G)** Two-sided Pearson's correlation analysis of TA measured by qTRAP and TERC abundance detected by RNAseq (N=951). Shaded area indicates the 95% confidence interval. Lower horizontal line indicates TA equivalent to 2% control and upper line is 10% control. Vertical line indicates median TERC level (N=951). TERC and TERT RNA expression data were downloaded from Cell Models Passport (version 20220624) [https://cellmodelpassports.sanger.ac.uk/downloads].

Figure S5

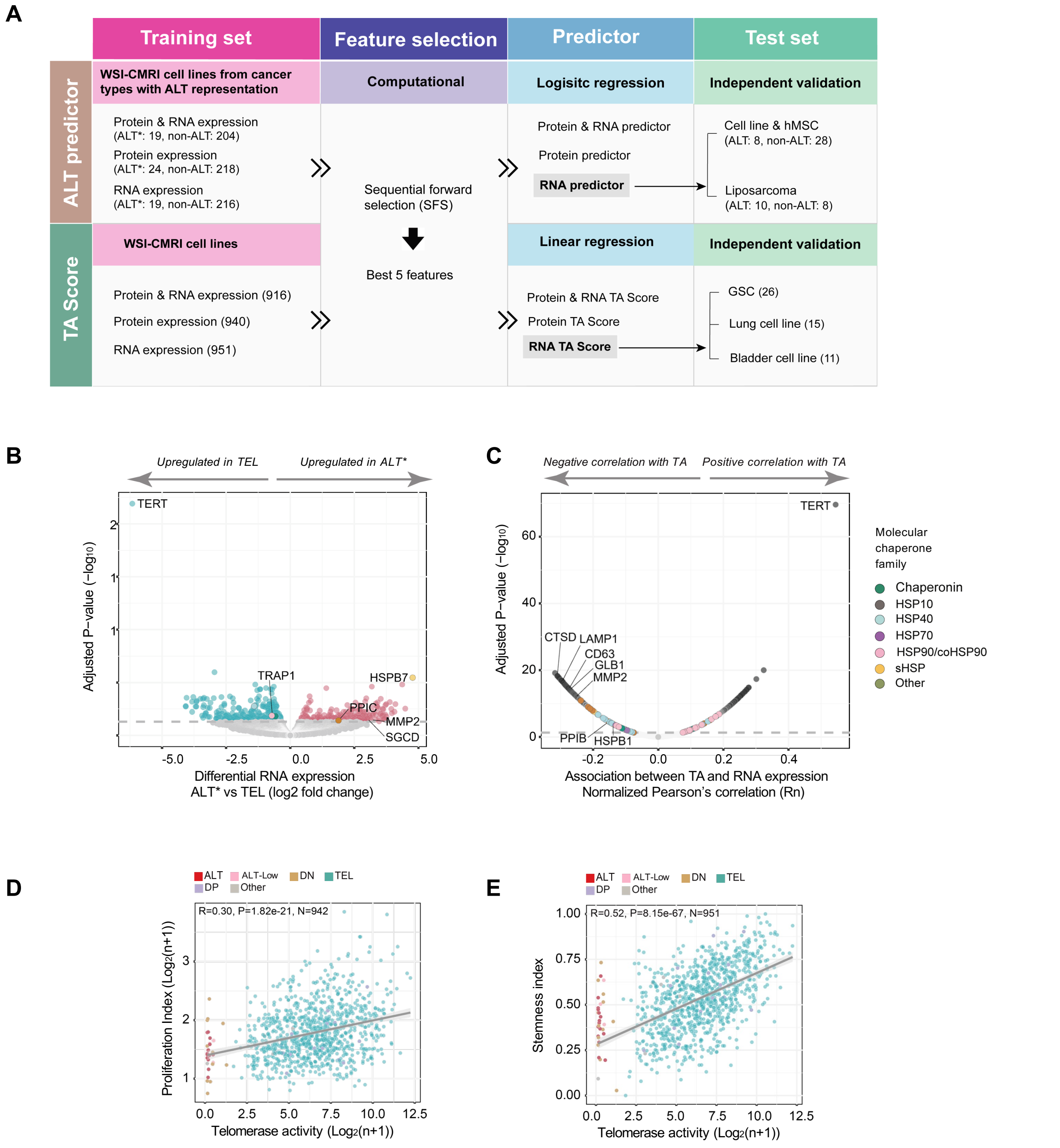

Figure S5. Machine learning methodology, gene expression analyses and phenotypic associations

(A) Methodology overview for derivation of algorithms for prediction of ALT (including ALT-Low) and TA levels. To improve class balance for ALT prediction, the cohort was down-sampled by excluding cancer types without ALT or ALT-Low cell lines. The number of cell lines in each analysis depended on coverage of the 'omic data sets and is shown in brackets. Sequential forward selection (SFS) was used to select the top five genes for input to generate the predictive algorithms. A class weight option was applied in logistic regression to further mitigate imbalance between the TMM groups. Algorithms were tested by cross-validation prediction. RNA-based algorithms were further validated on independent sample cohorts from previously published studies. **(B)** Differential RNA expression analysis of ALT versus TEL cell lines using tissue type as a covariate (N=918). **(C)** Two-sided normalized Pearson's correlation analysis of RNA intensity and TA with tissue types as a covariate (N=951). sHSP: Low molecular weight heat shock proteins. **(B-C)** Dashed line indicates adjusted P=0.05 determine by the Benjamini-Hochberg (BH) method using P-values from two-sided testing. Color scale indicates molecular chaperones from different chaperone families. Detailed results are in Supplementary Data 5. **(D)** Scatter plot showing the correlation between TA and Proliferation Index calculated from controls in the CellTitre-Glo Assay (N=942). Data for Proliferation Index is in Supplementary Data 1. **(E)** Scatter plot showing associations between TA and Stemness Index calculated from RNA expression data (Supplementary Data 5). Shaded area in **(D-E)** indicates the 95% confidence interval (N=951).

Figure S7

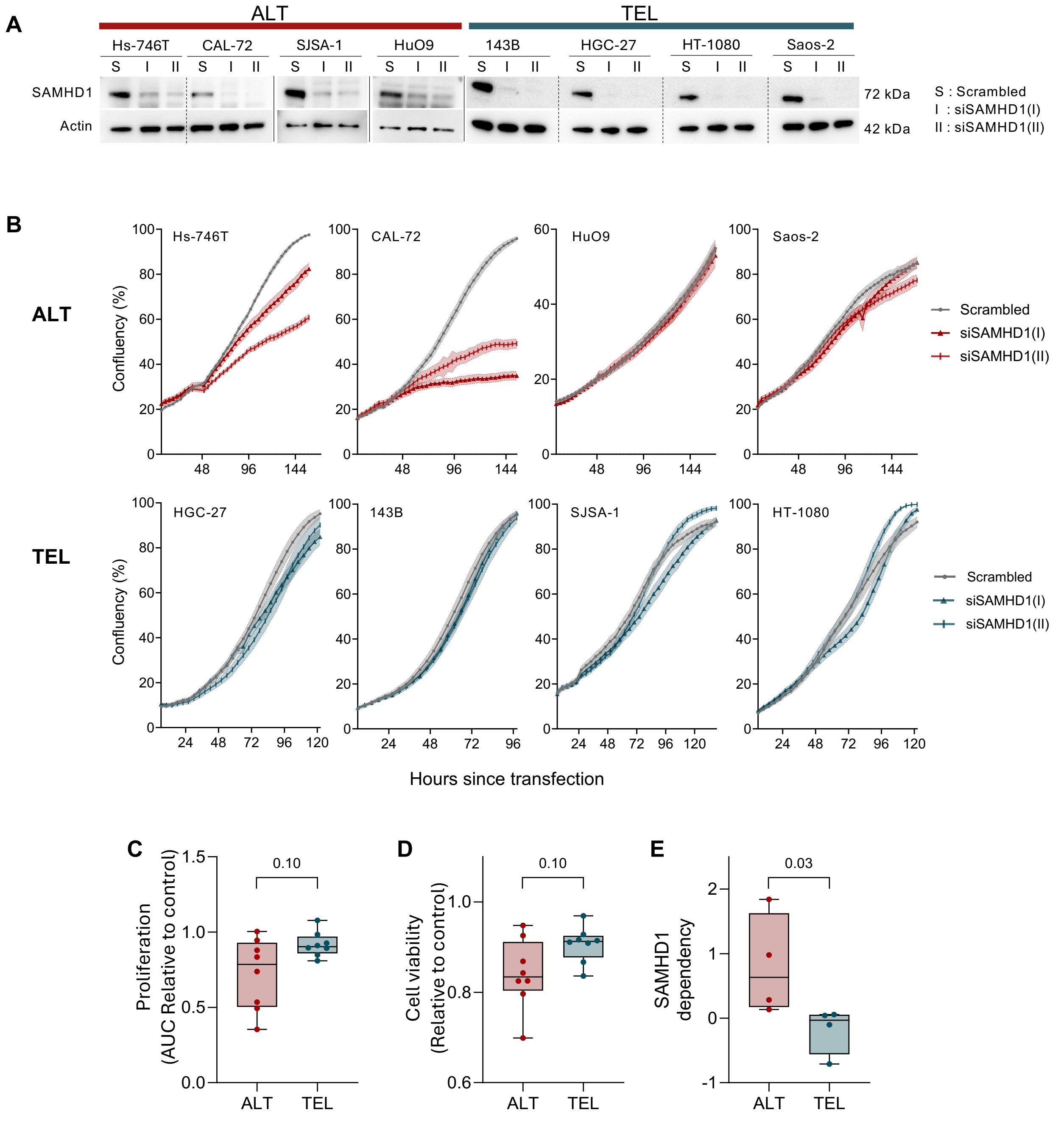

Figure S6. Validation of SAMHD1 gene dependency results

Cell lines (n=8; 4 ALT and 4 TEL) were transfected with two different siRNAs targeting SAMHD1 (siSAMHD1(I) and siSAMHD1(II)) and a control oligonucleotide (Scrambled) control in parallel cultures. Two independent experiments were performed with each cell line. **(A)** Western blot from a representative experiment showing siRNA-mediated suppression of SAMHD1 72 hours after transfection with siRNA. Similar knockdown was demonstrated by an independent Western blot. **(B)** Proliferation of siRNA-transfected cells from a representative experiment with n=8 cell lines, with each cell line assayed in triplicate. Data points show the average confluency with the shaded area indicating the standard error of the mean from triplicate wells imaged every four hours using an Incucyte® ZOOM system. **(C-D)** Summary of cell proliferation (C) and viability (D) for 8 cell lines (n=4 ALT and n=4 TEL) transfected with either siSAMHD1(I) or siSAMHD1(II). Proliferation was measured as the area under the curve (AUC) from cultures grown for 7 days and cell viability was measured in parallel cultures using CellTiter-Glo® 72 hours after siRNA transfection. Data points are average values from triplicate assays of each cell line (n=8; 4 ALT and 4 TEL) transfected with either siSAMHD1(I) or siSAMHD1(II). Results were normalized to the Scrambled control. **(E)** Gene dependency scores for SAMHD1 in high-throughput whole genome CRISPR-Cas9 knock-out screens of the 8 cell lines (n=4 ALT and n=4 TEL cell lines) used in the siRNA knockdown experiments. A higher dependency score indicates higher gene essentiality. Boxplots in (C-E) show 25th and 75th percentiles (box edges), the median (horizontal line), and 1.5x interquartile range as error bars. P-values were determined from comparison of ALT (n=4) and TEL (n=4) cell lines using the two-sided Wilcoxon rank-sum test.

Figure S7

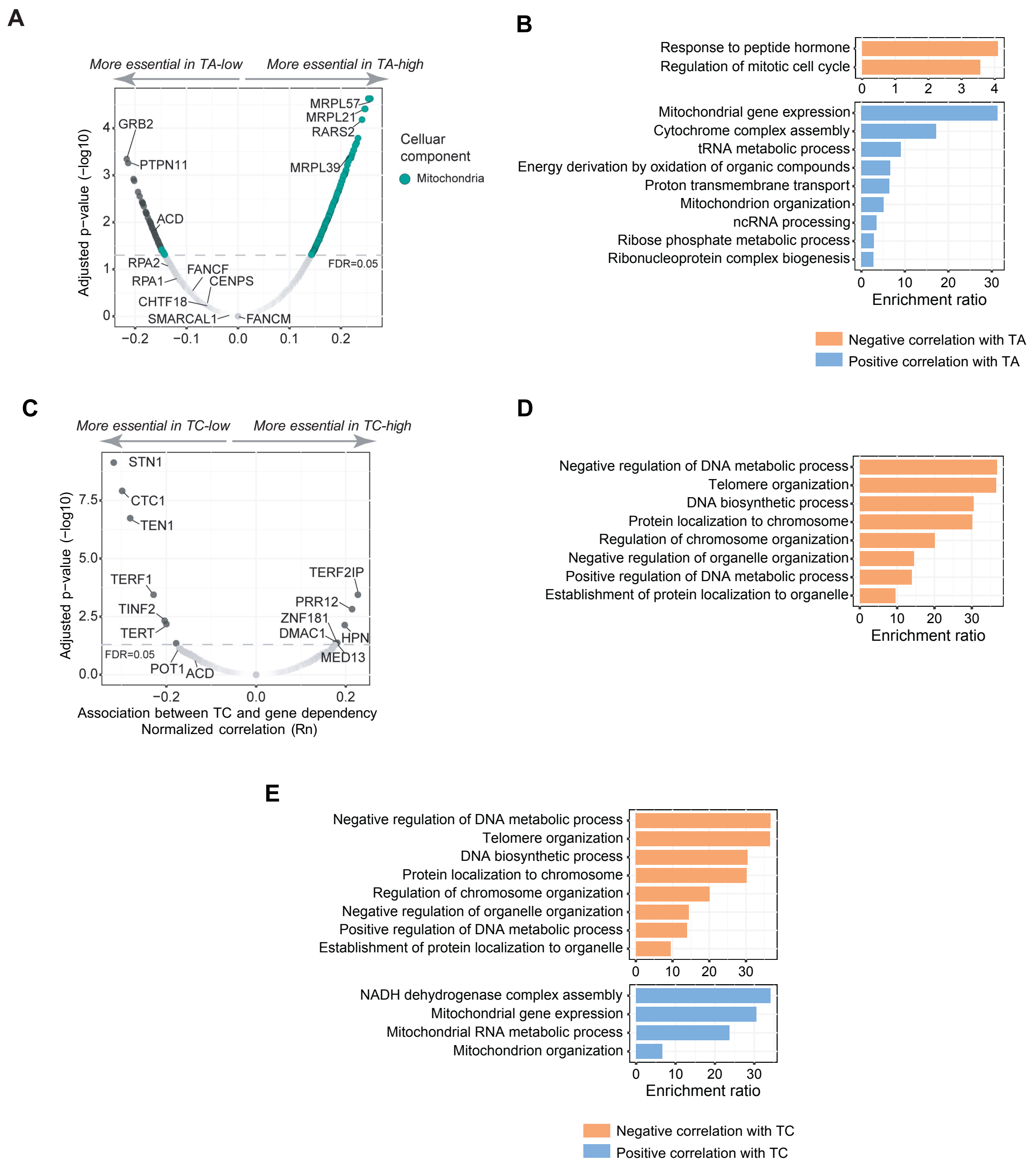

Figure S7. Gene dependencies associated with TMM

(A) Two-sided normalized Pearson's correlation analysis of TA and gene dependency scores in non-ALT cell lines (N=525) using tissue lineage as a covariate. **(B)** Gene Ontology (GO) terms (Biological Processes) from over-representation analysis of preferentially essential genes (PEGs) that significantly correlate with TA in non-ALT cell lines in Figure S7A. FDR<0.05. **(C)** Two-sided Normalized Pearson's correlation analysis of TC and gene dependency scores in cell lines from all TMM groups using tissue lineage as a co-variate (N=540). **(D)** Over-representation analysis of PEGs from Figure S7C using GO Biological Processes where FDR<0.05. **(E)** GO terms (Biological Processes) from over-representation analysis of PEGs from Figure 7G showing significant correlation with TC in non-ALT cell lines. In (A) and (C), N=503, and the dashed line indicates adjusted P-value=0.05 determined by the Benjamini-Hochberg (BH) method using P-values from two-sided testing. Detailed results are in Supplementary Data 6.

Figure S8

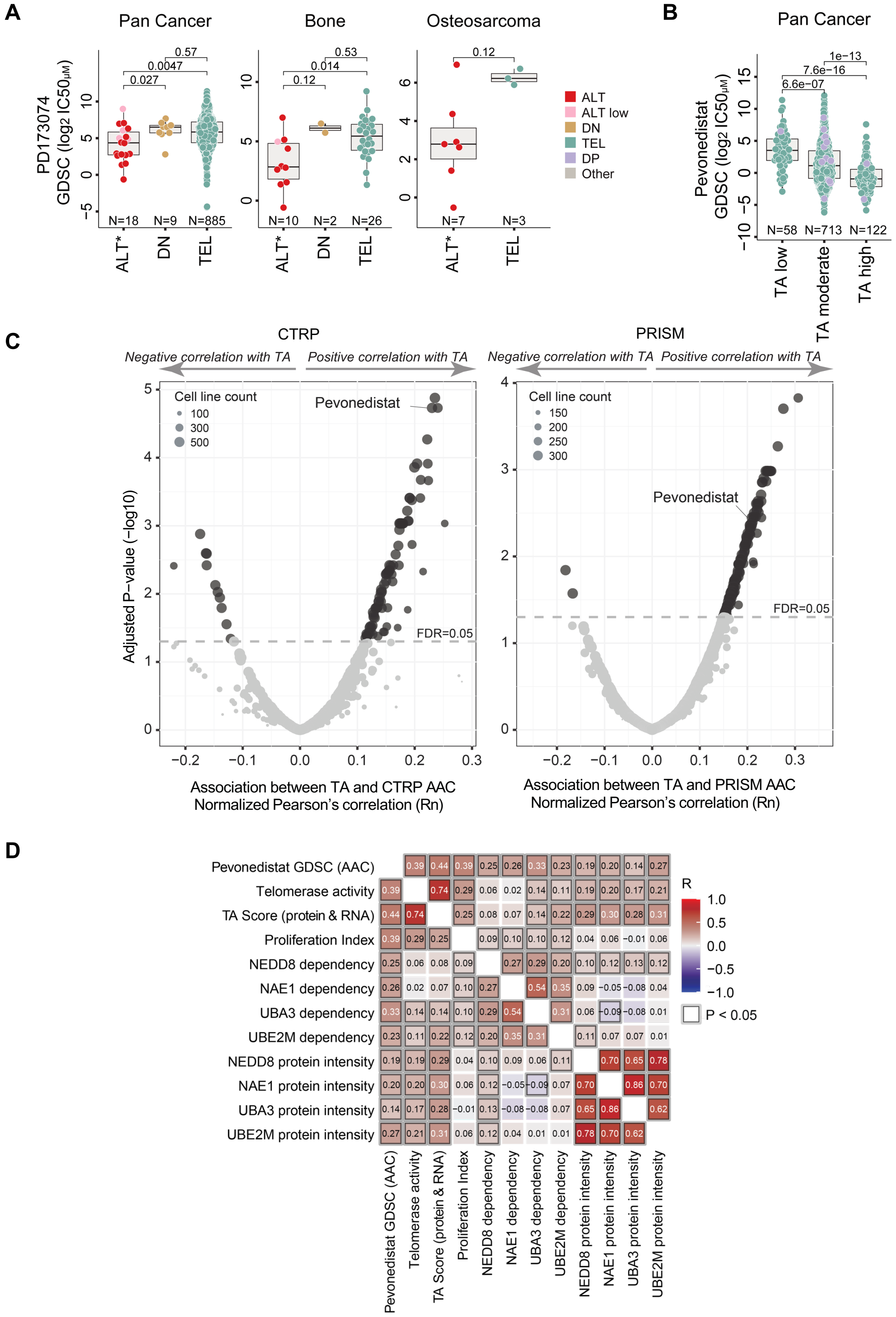

Figure S8. Correlations between TMM parameters and drug activity

(A) IC50 values for FGFR inhibitor PD173074, showing heightened response of ALT cell lines. ALT* includes ALT-Low cell lines. N indicates sample size. **(B)** IC50 values for Pevonedistat in cell lines with varying levels of TA. TA low: TA in the range of 2%-10% control; TA moderate: TA in the range of 10%-500% control; TA high: TA>500% control. N indicates sample size. **(C)** Two-sided normalized Pearson's correlation between TA and drug response using area above the curve (AAC) values from CTRP (version CTRPv2_2015) (N=544 drugs, N=575 cell lines) and PRISM data sets (version PRISM_2020) (N=1437 drugs, N=344 cell lines). Tissue type normalization was applied. Results are shown for drugs tested on at least five cell lines. Dot size indicates the number of cell lines tested with each drug. Dashed line indicates the adjusted P-value determined by the BH method. **(D)** Correlation matrix showing relationships between Pevonedistat, TA, gene dependency, and expression of proteins in the Pevonedistat target pathway. Box plots in (A-B) show the 25th and 75th percentiles (box edges), median (horizontal line), and error bars defining 1.5x the interquartile range. P-values were determined using the two-sided Wilcoxon rank-sum test. Detailed results, including sample size for each drug, are in Supplementary Data 7 and Source Data.

Source data for Figure S1A

The edges of the image were cropped for presentation purposes.

Final figure

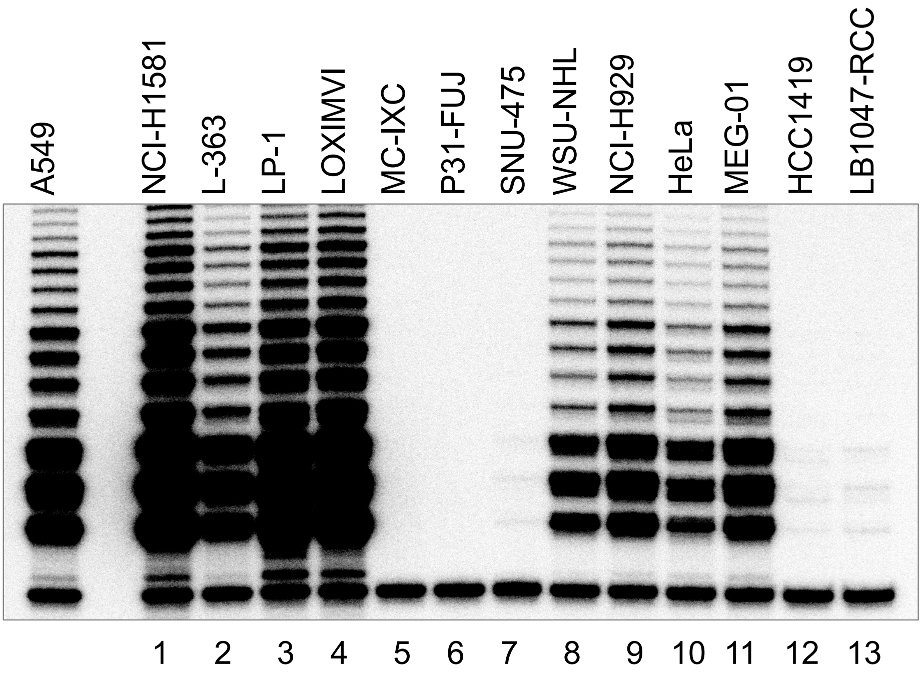

Uncropped image

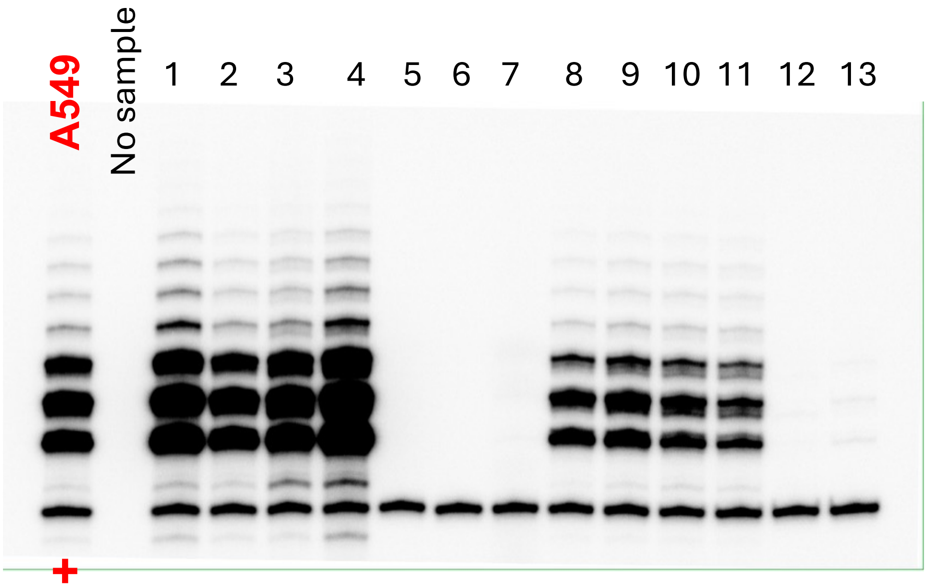

Source data for Figure S2B

The image was rearranged for presentation purposes

Final figure

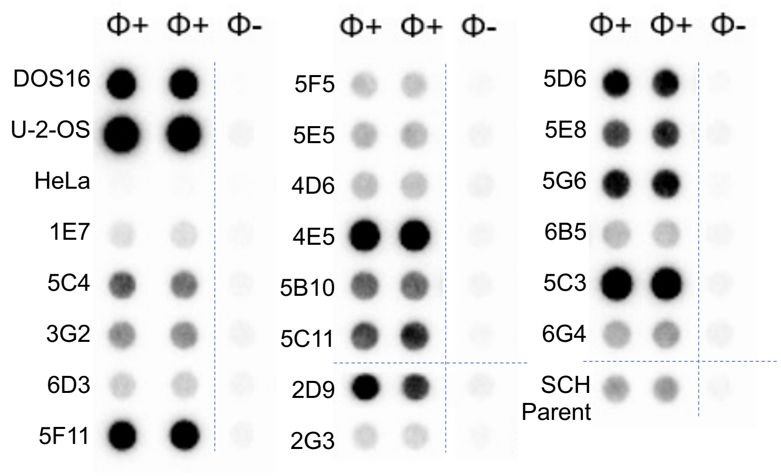

Unprocessed image

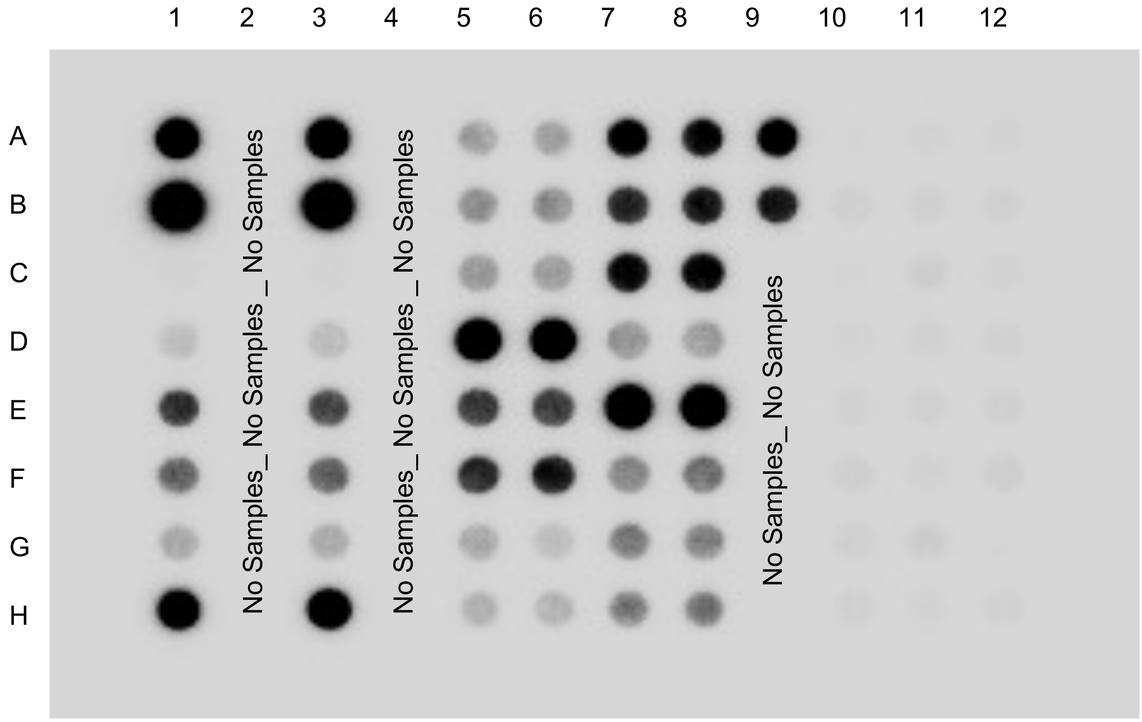

Sample manifest

	1	2	3	4	5	6	7	8	9	10	11	12
A	DOS16		DOS16		5F5	5F5	5D6	5D7	2D9	DOS-16	5F5	5D6
B	U-2-OS		U-2 -OS		5E5	5E5	5E8	5E9	2D9	U-2 OS	5E5	5E8
C	HeLa		HeLa		4D6	4D6	5G6	5G7		HeLa	4D6	5G6
D	1E7		1E7		4E5	4E5	6B5	6B6		1E7	4E5	6B5
E	5C4		5C4		5B10	5B10	5C3	5C4		5C4	5B10	5C3
F	3G2		3G2		5C11	5C11	6G4	6G5		3G2	5C11	6G4
G	6D3		6D3		Excluded	Excluded	Excluded	Excluded		6D3	2D9	Excluded
H	5F11		5F11		2G3	2G3	SCH PARENT	SCH PARENT		5F11	2G3	SCH PARENT
					ϕ+						ϕ-	

Source data for Figure S2C

Figure S2C is a composite image from 5 gels that were cropped and rearranged for presentation purposes.

Final figure

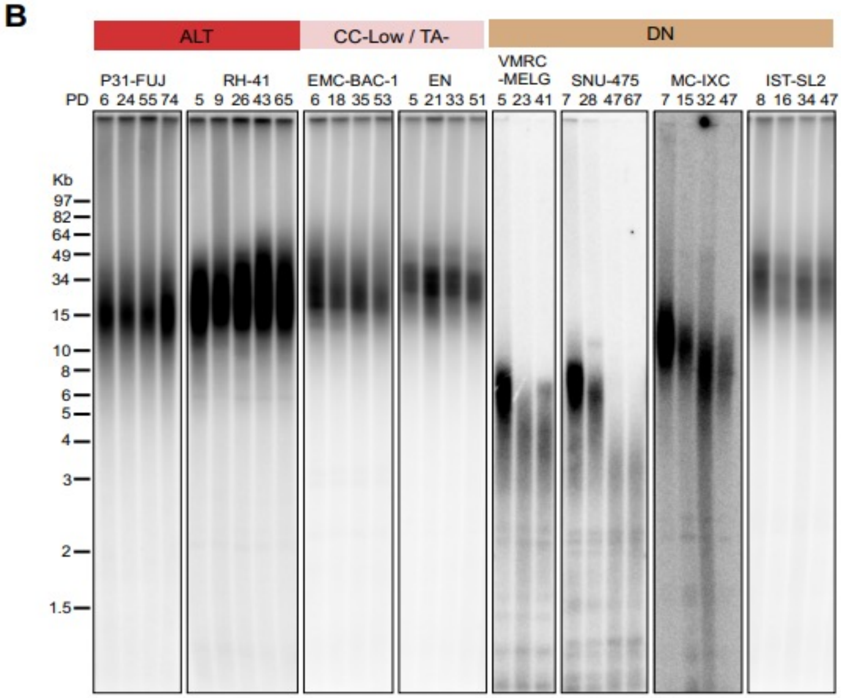

Uncropped images

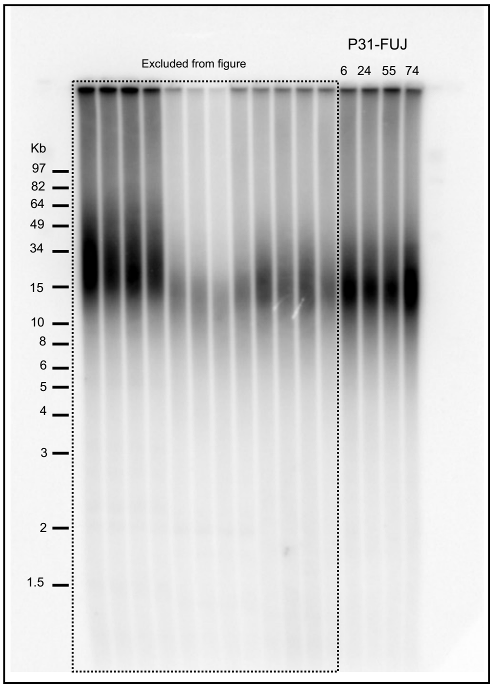

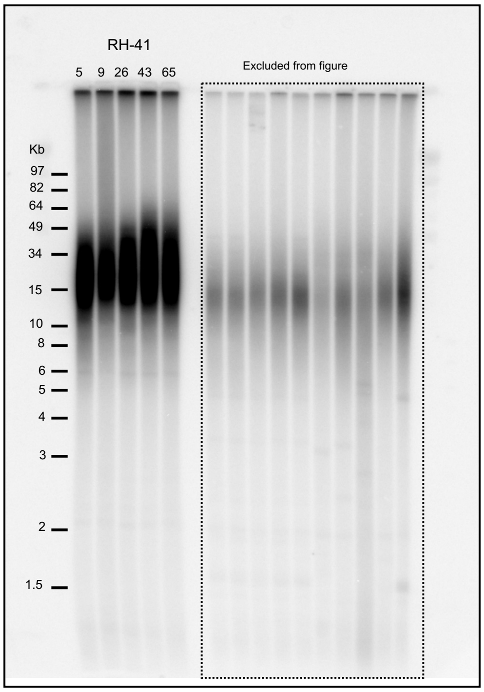

Source data for Figure S2C continued

Figure S2C is a composite image from 5 gels. The images from 5 gels were cropped and rearranged for presentation purposes.

Uncropped images

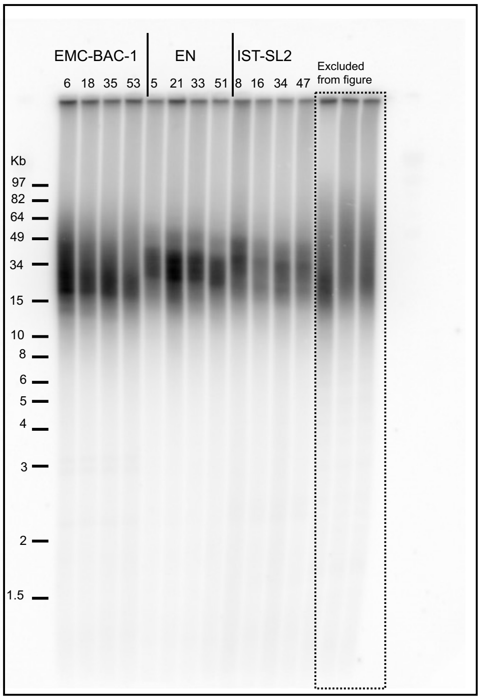

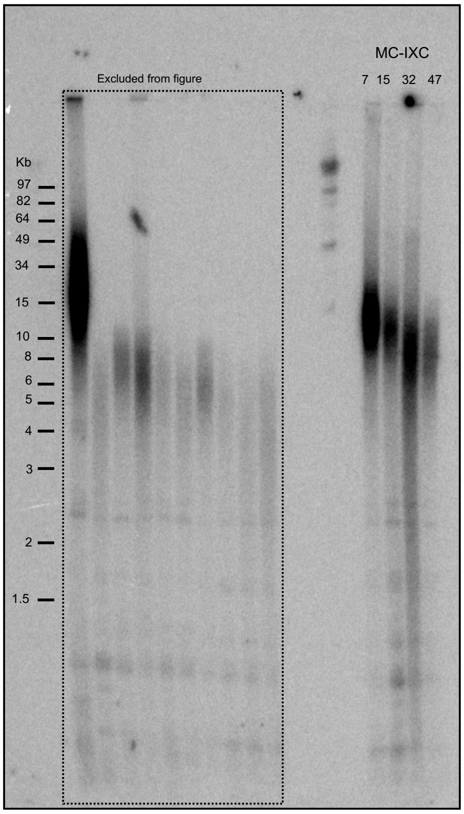

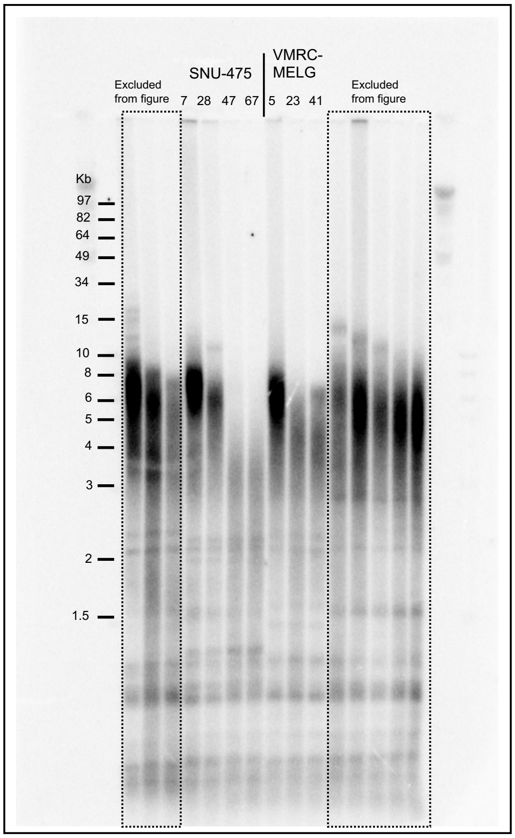

Source data for Figure S4D

The final Western blot image is a composite of representative non-ALT cell lines assayed across 3 gels. U-2-OS and HeLa were included on all gels as controls. The membranes were cut into 3 strips for simultaneous incubation with different antibodies (ATRX, DAXX and Actin). Images were cropped and rearranged for presentation purposes. ALT samples excluded from these gels are shown in Figure 4A.

Final figure

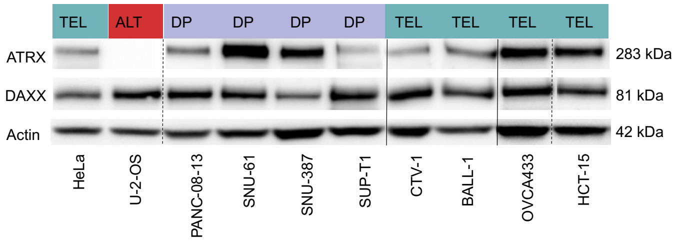

Uncropped images

ATRX

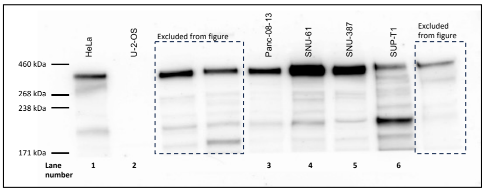

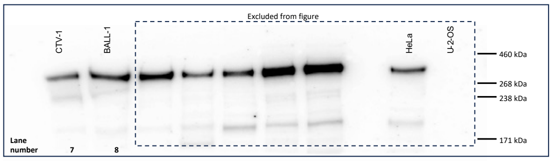

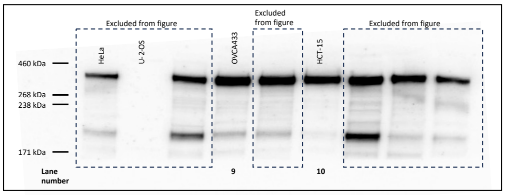

DAXX

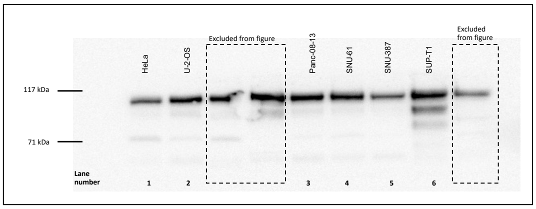

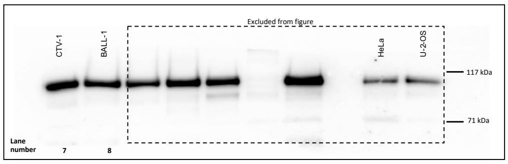

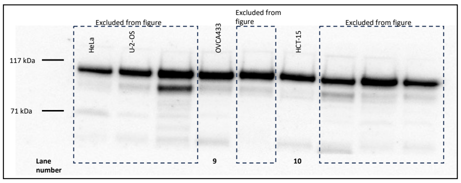

ACTIN

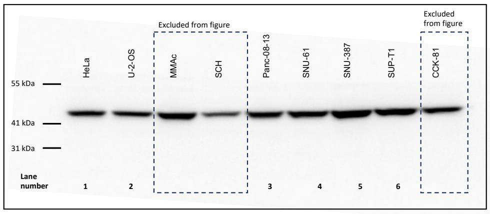

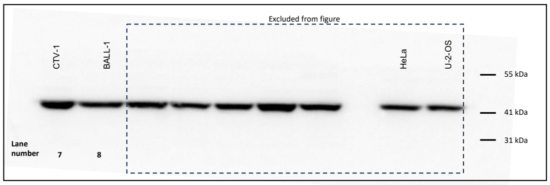

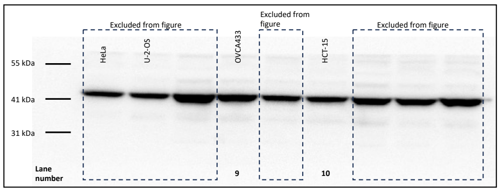

Source data for Figure S6A

The Western blot is a composite 4 gels. The membranes were cut into 2 strips for simultaneous incubation with SAMHD and Actin antibodies.

Final figure

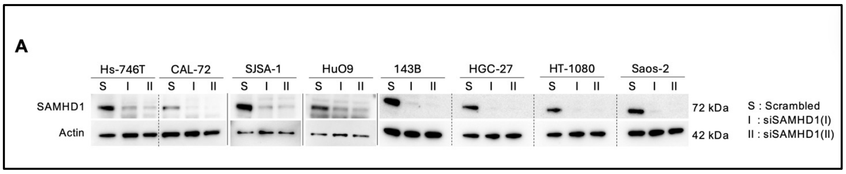

Uncropped images

SAMHD1

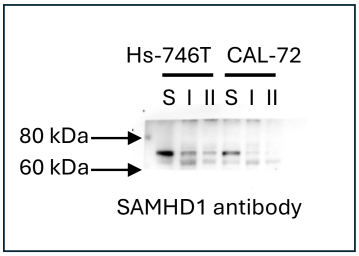

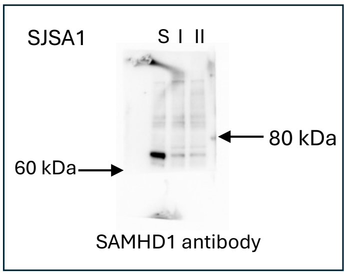

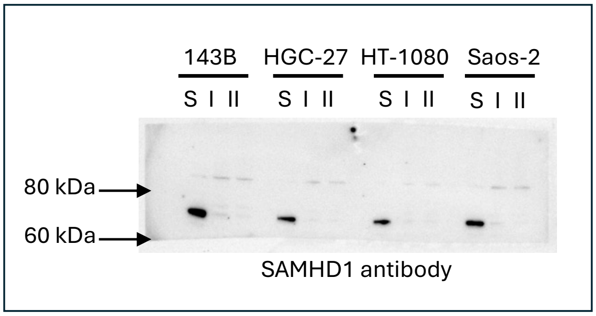

ACTIN

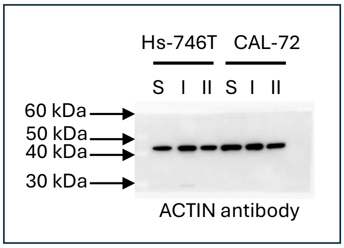

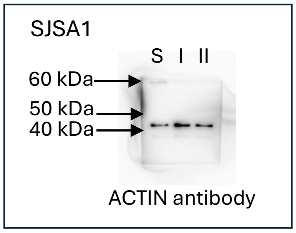

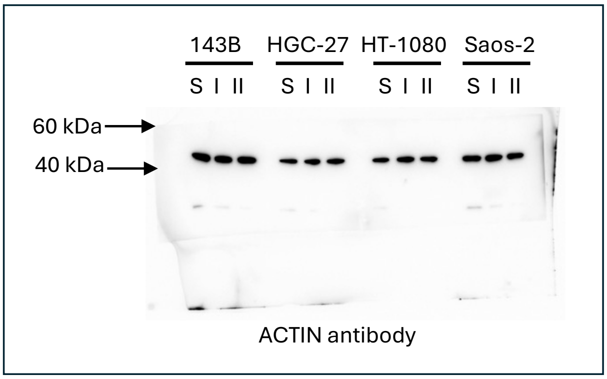

Supplement: Supplementary file 11 — Figure S1-S8 [file 41467_2025_67190_MOESM11_ESM.pdf]
